# Effect of bentonite as a soil amendment on field water-holding capacity, and millet photosynthesis and grain quality

**DOI:** 10.1038/s41598-020-75350-9

**Published:** 2020-10-26

**Authors:** Junzhen Mi, Edward G. Gregorich, Shengtao Xu, Neil B. McLaughlin, Jinghui Liu

**Affiliations:** 1 National Outstanding Agriculture Research Talents and Innovation Team, Inner Mongolia Agricultural University, Hohhot, 010019 Inner Mongolia China; 2 Central Experimental Farm, Agriculture and Agri-Food Canada, Ottawa Research and Development Centre, Ottawa, ON K1A 0C6 Canada; 3 Agricultural Environment and Resources Institute, Yunnan Academy of Agricultural Sciences, Kunming, 650205 Yunnan China

**Keywords:** Drought, Agroecology

## Abstract

A field experiment was conducted in a semi-arid region in northern China to evaluate the effects of bentonite soil amendment on field water-holding capacity, plant available water, and crop photosynthesis and grain quality parameters for millet [*Setaria italic* (L.) Beauv.] production over a 5-year period. Treatments included six rates of bentonite amendments (0, 6, 12, 18, 24 and 30 Mg ha^−1^) applied only once in 2011. The application of bentonite significantly (*P* < 0.05) increased field water-holding capacity and plant available water in the 0–40 cm layer. Bentonite also significantly (*P* < 0.05) increased the emergence rate, above-ground dry matter accumulation (AGDM), net photosynthesis rate (Pr), transpiration rate (Tr), soil and plant analysis development (SPAD) and leaf water use efficiency (WUE). It also increased grain quality parameters including grain protein, fat and fiber content. Averaged over all the years, the optimum rate of bentonite was 24 Mg ha^−1^ for all plant growth and photosynthesis parameters except for grain quality where 18 Mg ha^−1^ bentonite had the greatest effect. This study suggests that bentonite application in semi-arid regions would have beneficial effects on crop growth and soil water-holding properties.

## Introduction

Arable land is limited and loss of productivity by soil degradation poses a threat to food security in many areas. Environmental factors including moisture, temperature, soil quality and frost-free growing period, affect plant growing systems and crop yield, and play an important role in agriculture production^1,2^. Natural rainfall often cannot meet the crop requirements for water in arid and semi-arid regions^3^. Drought is one of the most critical factors affecting agricultural production in semi-arid rain-fed regions under a changing climate^4^. It is one of major factors influencing crop loss worldwide, reducing crop yields by up to 50% each year^5,6^. By 2050 climate change is estimated to lead to a global decline of crop production by 9%^7^. In addition, drought events are predicted to become more frequent in the future with global warming^8^. Therefore, adopting appropriate approaches to reduce the adverse effects of climate change on agriculture production is a priority.

Dryland farming accounts for more than 70% of the total arable land in northern China; low rainfall and poor soil quality are major constraints to agriculture production in this region^9^. The region along the Great Wall in Inner Mongolia in northern China is a semi-arid area and is characterized by low soil water-holding capacity which leads to low water use efficiency for crops. Therefore, water saving innovations are required to alleviate drought stress for sustainable development of agriculture in the arid and semi-arid regions where water is the primary limiting factor for agriculture production.

Millet is one of the main cultivated crops in China and is distributed in the arid and semi-arid regions of all provinces of northern China^10^ with a planted area of 8.61 × 10^5^ ha in 2017^11^. Virtually all millet production is rainfed and therefore drought is one of the most limiting factors. To cope with drought stress, different strategies including deficit irrigation, breeding new drought resistant plant varieties, and mulching are being practised^12–14^. More effective strategies are needed for dealing with water shortages in arid and semi-arid regions because current water management strategies are not able to ensure sustainability of agriculture production in these regions. Application of soil amendments which increase soil water-holding capacity is one strategy that is receiving considerable attention in agriculture research.

Photosynthesis characteristics play a fundamental role in physiological processes that affect plant carbon metabolism in cells and growth under harsh environmental conditions such as drought and heat^15,16^. Leaf structure and soil properties both affect photosynthesis, and drought decreases plant photosynthesis activity in leaves, and consequently, decreases crop growth and quality^17^. Addition of a superabsorbent polymer to soil has been shown to retain water and nutrients that are released slowly as required by crop growth, thereby improving soil physical, chemical and biological properties, and increasing soil productivity^18,19^. Studies have shown that application of these types of soil amendments can improve the available soil water-holding capacity, reduce the evapotranspiration, increase the water and fertilizer use efficiency in crops and increase crop emergence, growth and yield^20,21^. They also have been shown to have potential for improving crop photosynthesis characteristics in the field^22^. This is attributed to improvement in leaf stomatal conductance and CO_2_ assimilation rate by increased transpiration rate^23–25^.

Bentonite is a natural soil amendment which is very abundant in China, with over 8.0 Pg of proven reserves^26^. At present bentonite is widely used for adsorbing metal ions and dye, disposing radioactive wastes and purifying viral RNA and waste water due to its strong adsorption properties^27–30^. Furthermore, its use as a soil amendment may be an effective approach for solving some of the agronomic/environmental problems related to drought stress and soil degradation in agricultural systems. In our previous paper, results showed that bentonite increased soil moisture and soil water storage, increased millet yield and improved water use efficiency^31^. Other research reported that clay as a soil amendment added to sandy soil can improve soil available water, soil organic carbon and soil potassium, and increase crop emergence and yield^32^. This was attributed to increase in soil aggregation which results in an improvement in soil porosity soil water-holding capacity, soil fertility and crop growth^33^. There are some studies on clay and polymer superabsorbent soil amendments used in agriculture, but little information is available about effects of bentonite as a soil amendment on crop photosynthesis characteristics and grain quality in agriculture production.

The objective of this study was to evaluate the effect of bentonite amendments on field water-holding capacity and plant available water in the 0–60 cm layer, emergence rate, above-ground dry matter accumulation, photosynthesis and grain quality parameters for millet production over five years in a semi-arid region. Preliminary data on a subset of the field measurements, emergence rate and above-ground dry matter accumulation were published for the first three years in the same field experiment in a Chinese journal and a non-peer reviewed conference proceedings^34,35^. The present paper covers a wider range of measurements including field water-holding capacity, plant available water, photosynthesis parameters and grain quality attributes to provide a more complete assessment of the research over five years. Meanwhile preliminary published data were cited to determine the changes in emergence rate and above-ground dry matter accumulation in the fourth and fifth year after application of bentonite.

## Materials and methods

### Experimental site and design

The field experiment was conducted from 2011 to 2015 in Yijianfang village (111°39′E, 39°57′N) of Qingshuihe County, Hohhot, Inner Mongolia, China. Mean annual rainfall is 365 mm and mean annual potential evaporation is 2577 mm. The experimental site is in the hilly gully region of the loess plateau in northeastern China, the soil genesis is loess, and the soil texture of the field is sandy loam. The initial soil chemical properties (Table 1) and climate data in the experimental site were provided in our previous paper^31^.

**Table 1:** Soil property data in the experimental site (data from Mi et al.^31^).

Property	Value
Texture	Sandy loam
Sand content (%)	72.8
Silt content (%)	13.4
Clay content (%)	13.8
Soil pH	8.0
Soil cation exchange capacity (cmol kg^−1^)	8.3
Soil bulk density (g cm^−3^)	1.42
Soil organic matter (g kg^−1^)	10.96
Total N (mg kg^−1^)	0.49
Total P (mg kg^−1^)	0.43

The experimental design was a randomized complete block with three replicates and included six treatments. Each plot was 6 m × 5 m. The treatments consisted of six rates of bentonite: 0, 6, 12, 18, 24 and 30 Mg ha^−1^. The bentonite was applied only one time in 2011 and was broadcast with fertilizer prior to seeding and mixed into the soil to depth of about 30 cm by cultivating. Tillage management included mouldboard plow followed by cultivating in the spring. Diammonium phosphate (DAP, 18-46-0) and urea (46-0-0) as starter fertilizer were applied each year at 225 and 75 kg ha^−1^ respectively; additional urea was applied at 150 kg ha^−1^ at approximately 60 d after sowing.

Bentonite composition and cost are given in Table 2. K_2_O was the only major plant nutrient in the bentonite. The soil was slightly alkaline, and the addition of CaO and MgO in the bentonite had only a small effect on the soil pH which ranged from 8.00–7.87.

**Table 2:** Bentonite composition and cos.

Bentonite composition (on a weight basis) and cost							
SiO_2_	Al_2_O_3_	Na_2_O	CaO	MgO	K_2_O	Fe_2_O_3_	Cost
73.20%	11.40%	0.31%	2.67%	1.05%	2.58%	0.29%	63 USD mg^−1^

Millet (variety: No. 5 Zhang) was planted at the beginning of May and harvested in the middle of September in each year. The seeding depth was 3–5 cm, row spacing was 25 cm, and planting density was 180,000–225,000 plants ha^−1^. On the same day as millet planting in the spring, an additional 300 millet seeds were planted by hand between two rows in each plot for measurement of emergence rate (ER) after 30 days; the plants were later removed after emergence counts. At maturity, a 1 m^2^ area (four rows of 1 m length) from each plot was randomly selected and harvested by hand to measure millet yield.

### Field and laboratory measurements

Duplicate undisturbed soil samples for field water-holding capacity were taken at depths of 0–10, 10–20, 20–40 and 40–60 cm at 90 d after sowing by the cutting ring method^36^ from 2013 to 2015. Undisturbed soil samples in the cutting rings were placed in 4.0–4.8 cm of water until they were saturated. The second set of soil samples was air-dried, passed through 1-mm mesh screen and placed in rings of the same size. The rings with undisturbed saturated soil were covered and placed on top of the rings with air-dry soil for 8 h. The rings with undisturbed soil were then taken to be at field capacity which was determined by oven drying a sub-sample.

Plant available water (PAW) from 2013 to 2015 was determined by field water-holding capacity (FC) and wilting point (WP) (− 1500 kPa soil water tension). Plant available water was calculated by equation: PAW = FC-WP^37^.

Percent germination was determined in the laboratory by placing 100 millet seeds on a damp filter paper for 15 days at room temperature.

Above-ground dry matter accumulation (AGDM) of the millet plants was measured by gravimetric method at 50, 70, 90, 110 and 130 d after sowing to monitor the bentonite effect on plant growth over the growing season. Ten plants were retrieved from each plot and oven-dried at 105 °C for 30 min, and then at 75 ºC for 24 h until constant weight. The equivalent field area for the ten plants was 0.5 m^2^.

Net photosynthesis rate (Pn) and transpiration rate (Tr) from 2013 to 2015 were measured at 90 d after planting with a Li 6400 portable photosynthesis system equipped with a 18 cm^3^ prismatic leaf chamber (Li 6400, Licor, Lincoln, NE, USA) as described in Arbona et al.^22^. Ten randomly selected flag leaves in each plot were measured. All the determinations were performed under the constant air flow rate (500 μmol s^−1^), and conducted between 9:00–11:00 am when the temperature was 24 ± 2 °C on a sunny day. Leaf water use efficiency (WUE) was calculated by equation: WUE = Pn/Tr.

SPAD was measured at 90 days after planting (around heading stage) from 2013 to 2015 with a Soil and Plant Analysis Development meter (SPAD-502, Konica Minolta, Tokyo, Japan). It was also performed on ten (different) flag leaves per plot similar to those for the net photosynthesis rate measurements. Three positions selected per flag leaf were measured and then the average was calculated.

Mature millet seeds were ground into flour for measurement of total protein, fat and fiber from 2011 to 2015.

Total grain protein content was measured using the Kjeldahl method^38,39^. A 1.0 g sample was digested with 20 ml H_2_SO_4_ at 300 °C until the digestate was clear. After cooling, 20 ml distilled water was added, agitated, and then further diluted to 100 ml with distilled water. Then a sample of 10 ml of the solution was distilled with 10 ml of 12% H_3_BO_3_ and 10 ml of 40% NaOH solution for 7 min, and titrated with 0.5 N HCl.

Total fat content in the grain was measured with a fat analyser using the Soxhlet extractor method^40,41^. A 3 g sample wrapped with filter paper was alternately steeped in the diethyl ether, and washed with water at 70–80 °C until no oil was visible in the wash water. After the diethyl ether was volatilized, the samples were dried under vacuum.

Total fiber content in the grain was measured with a gravimetric method^42^. After extracting total fat, a 1.0 g dried sample was extracted with 200 ml of 1.25% H_2_SO_4_ and 1.25% NaOH solution at 100 °C for 1.5 h. Samples were then steeped in acetone for 10 min, oven-dried at 105 °C until constant weight and then fired at 550 ºC until constant weight.

The field water-holding capacity, plant available water, photosynthesis parameters were measured from 2013 to 2015 to determine their changes three to five years after the application of bentonite amendment. In this paper the last two-years of data on AGDM and ER are shown in Figs. 4 and 3; the data for the first three years were previously published^34,35^. We combined published preliminary data from the same experiment with the present data to determine the changes in AGDM and ER over the five years following the single application of bentonite amendment in 2011.

### Data analysis

The FWC, PAW, ER, AGDM, SPAD, photosynthesis parameters (Tr, Pn, SPAD and WUE) and grain quality for each treatment were calculated by averaging three replicates for each plot, i.e. averaging subplot measurements. The normality and homogeneity of variances were checked using Shapiro–Wilk and Bartlett test before performing an analysis of variance (ANOVA). A two-way linear models ANOVA was performed to analyze the differences in Pn, Tr, SPAD, WUE, protein, fat, fiber, ER and AGDM among bentonite amendments. Statistical analyses were performed with SAS Ver. 9.3 software package for Windows (SAS Institute Inc., Cary, NC, USA). Differences among bentonite amendment means were determined using the linear model with Tukey’s multiple comparisons at 5% level. Pearson’s correlations were performed to determine the relationships among all the parameters. Data were plotted using Sigmaplot 10.0 (Systat Software, Inc. San Jose, CA,USA).

## Results

### ANOVA of measured parameters

The ANOVA for different measurements (Tables 3 and 4) showed that bentonite treatment (T), day (D) and year (Y) had a highly significant (*P* < 0.01) effect on emergence, AGDM, Pn, Tr, SPAD, WUE, protein, fat and fiber. The interaction of T*Y had a significant (*P* < 0.05) effect on all of the measured parameters except Tr, WUE and protein. The interaction of T*D, D*Y, T*Y and T*D*Y had a highly significant (*P* < 0.01) effect on AGDM.

**Table 3:** ANOVA of effect of bentonite treatments (T) and year (Y) on photosynthetic parameters.

Factors	DF	Pn		Tr		SPAD		WUE	
		F value	*P*	F value	*P*	F value	*P*	F value	*P*
T	5	135.97	< 0.001***	11.19	< 0.001***	78.9	< 0.001***	10.08	< 0.001***
Y	2	50.35	< 0.001***	87.66	< 0.001***	95.09	< 0.001***	73.64	< 0.001***
T*Y	10	7.65	< 0.001***	1.73	NS	2.38	0.014*	0.47	NS

*and ***Significant at 0.05 and 0.001 level respectively; NS means not significant. Pn: net photosynthetic rate; Tr: transpiration rate; SPAD: leaf soil plant analysis development; WUE: leaf water use efficiency. Pn, Tr, SPAD, and WUE were measured at 90 d after sowing over the last three years, 2013 to 2015.

**Table 4:** ANOVA of effect of bentonite treatments (T), day (D) and year (Y) on emergence rate, AGDM and grain quality parameters, protein, fat and fiber.

Factors	DF	Protein		Fat		Fiber		Emergence		AGDM	
		F value	*P*	F value	*P*	F value	*P*	F value	*P*	F value	*P*
T	5	21.22	< 0.001***	134.22	< 0.001***	428.83	< 0.001***	114.34	< 0.001***	758.47	< 0.001***
D	4									45,034.1	< 0.001***
T*D	20									64.62	< 0.001***
Y	4	7.66	< 0.001***	109.83	< 0.001***	294.75	< 0.001***	24.08	< 0.001***	3786.67	< 0.001***
T*Y	20	0.41	NS	13.34	< 0.001***	43.95	< 0.001***	3.75	< 0.001***	22.15	< 0.001***
Y*D	16									611.36	< 0.001***
T*Y*D	80									8.7	< 0.001***

*** Significant at 0.001 levels; NS means not significant. Protein, Fat, Fiber, Emergence and AGDM were measured over five years, 2011 to 2015.

### Field water holding capacity (FC)

**Figure 1:**
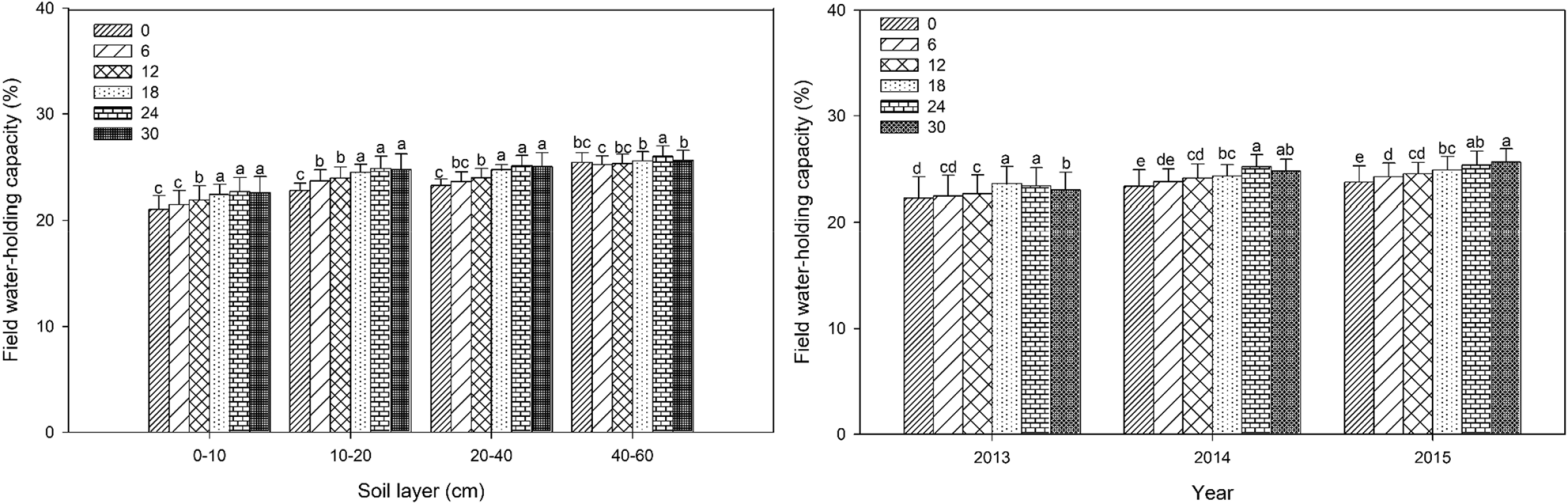
Field water-holding capacity for different rates of bentonite measured at 90 d after sowing in the 0–60 cm soil layer from 2013 to 2015. Left panel: mean of 2013 to 2015 by soil layer; right panel: mean of 0–60 cm soil layer by year. Bars within the same year or the same layer and with the same letters are not significantly different at the 0.05 level of probability. Error bars are standard deviations.

All bentonite amendments significantly (*P* < 0.05) increased the FC compared with control without bentonite at 0–40 soil layers except for 6 mg ha^−1^ at 0–10 and 20–40 cm layers in 2013. None of the bentonite amendments had an effect at the 40–60 cm layer (Fig. 1). In 2013, 2014 and 2015, FC for bentonite treatments was significantly increased by up to 9%, 11% and 12%, respectively. Improvements in FC for all bentonite treatments ranged from 1 to 9% for the 0–10 cm layer, from 1 to 12% for the 10–20 cm layer, and from 0 to 11% for the 20–40 cm layer. The effect of bentonite amendment addition to soil showed a trend of increased FC over time at deeper layers. The trend was similar for all soil layers for three years except for the 40–60 cm layer: averaged over three years, 24 Mg ha^−1^ treatment had the greatest effect.

### Plant available water (PAW)

**Figure 2:**
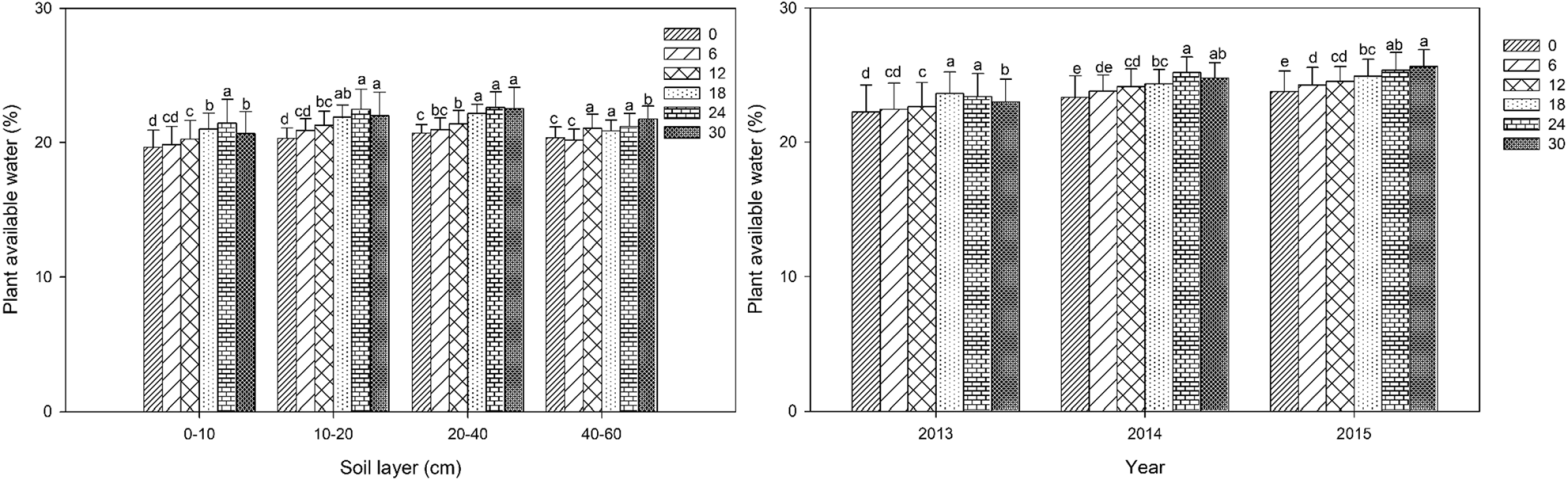
Plant available water with different rates of bentonite amendment at 90 d after sowing in the 0–60 cm soil layer from 2013–2015. Left panel: mean of 2013 to 2015 by soil layer; right panel: mean of 0–60 cm soil layer by year. Bars within the same year or the same layer and with the same letters are not significantly different at the 0.05 level of probability. Error bars are standard deviations.

All bentonite amendments significantly (*P* < 0.05) increased PAW compared with control in 0–40 cm layers (Fig. 2). Bentonite amendments had no effect in 40–60 cm layer. In 2013, 2014 and 2015, observed increases in PAW were up to 6%, 10% and 10%, respectively. Improvement in PAW ranged from 1–9% for the 0–10 cm layer, from 4–10% for 10–20 cm, from 1–9% for 20–40 cm and from 1–9% for the 40–60 cm layers. The amendment effect showed a trend for increasing effect over time. The trend for all layers was similar to FC from 2013–2014: averaged over three years, the treatment with 24 mg ha^−1^ bentonite had the largest effect.

### Emergence rate (ER)

**Figure 3:**
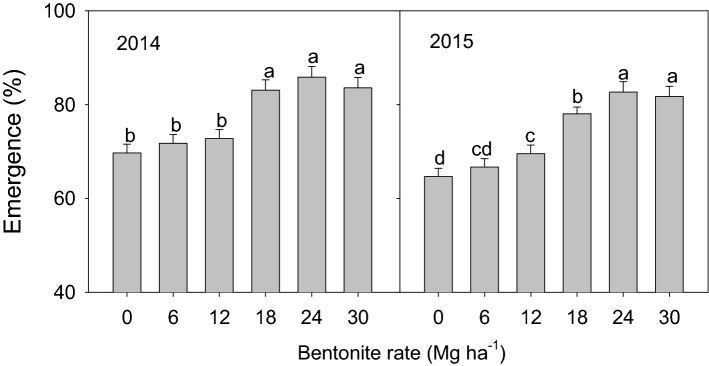
Millet emergence (%) at 30 days after sowing with different rates of bentonite in 2014 and 2015. Bars within the same year and with the same letters are not significantly different at the 0.05 level of probability. Error bars are standard deviations.

The effect of bentonite on ER in 2014 and 2015 is presented in Fig. 3; emergence data from the first three years of the same experiment were presented in our previous paper^31^. The present additional data combined with our earlier data now show that bentonite and interaction of bentonite treatment and time had a significant (*P* < 0.05) effect for ER over five years and more importantly, that there was greater effect in later years. The increase in ER for all bentonite treatments over the control with no bentonite ranged from 2–16% and 2–18% in 2014 and 2015, respectively.

### Aboveground dry matter (AGDM)

**Figure 4:**
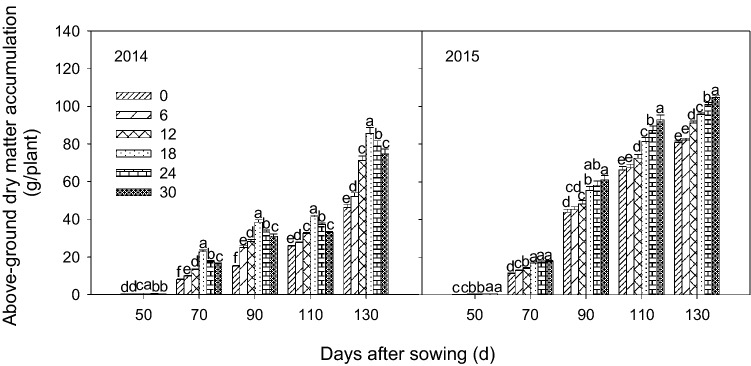
Above-ground dry matter accumulation with different rates of bentonite at different days after sowing in 2014 and 2015. Bars in the same year and with the same letter are not significantly different at the 0.05 level of probability. Error bars are standard deviations.

AGDM data at different days after planting from the first three years of the same experiment were presented in our previous paper^32^. For all bentonite treatments, AGDM was significantly increased by up to 187% and 111% in 2014 and 2015, respectively compared with the control without bentonite (Fig. 4). The greatest percentage differences among all treatments occurred at 70 d after sowing in the first four years; in 2015 the difference was greatest at 50 d after sowing, likely due to the very low rainfall over the growing season. In the first four years, 18 and 24 mg ha^−1^ treatment had significantly (*P* < 0.05) higher AGDM than the other treatments while, in 2015, the AGDM over growing season was the largest for 30 mg ha^−1^ treatment.

### Photosynthesis characteristics (Pn, Tr, SPAD, and WUE)

**Figure 5:**
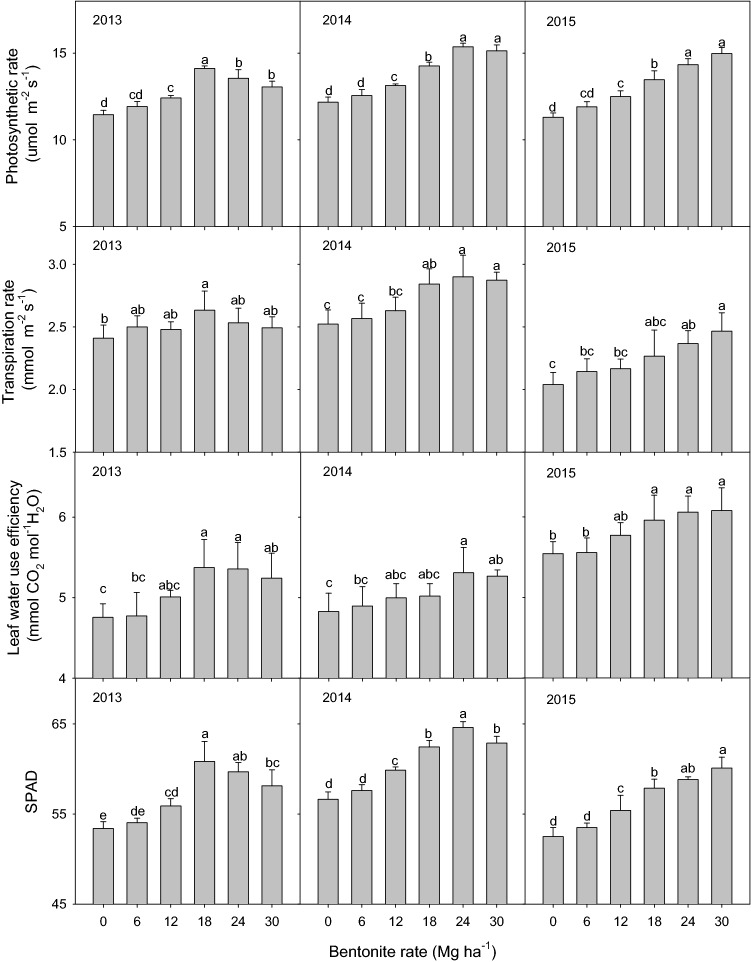
Millet photosynthesis parameters with different rates of bentonite at 90 d after sowing from 2013 to 2015. Bars within the same photosynthesis parameter, same year and with the same letters are not significantly different at the 0.05 level of probability. Error bars are standard deviations.

Bentonite significantly (*P* < 0.05) increased Pn, Tr, SPAD and WUE compared to control without bentonite from 2013 to 2015 (Fig. 5). Increases in Pn, Tr, SPAD and WUE were 3–32%, 2–21%, 1–15% and 0–13% compared with control without bentonite in all three years. All of the photosynthesis parameters showed a similar trend: in the third year (2013), 18 mg ha^−1^ bentonite had the greatest effect; and averaged over three years, 24 mg ha^−1^ bentonite had the greatest effect.

### Grain quality

**Figure 6:**
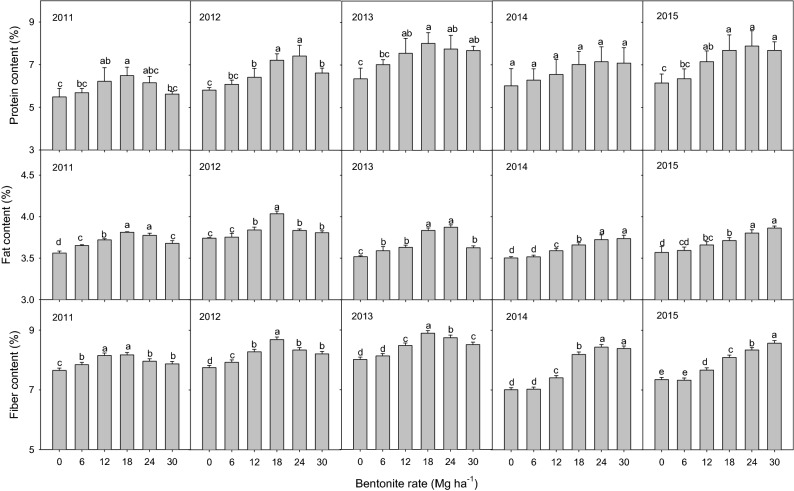
Millet grain quality parameters with different rates of bentonite from 2011 to 2015. Bars within the same grain quality parameter, same year and with the same letters are not significantly different at the 0.05 level of probability. Error bars are standard deviations.

Grain protein, fat and fiber all increased with increasing bentonite rates in all five years (Fig. 6). Exceptions were in 2014 when the effect on grain protein was not significant and in the last three years, when there were no significant (*P* > 0.05) differences in grain fiber between 0 and 6 mg ha^−1^ bentonite. In the first three years, 18 and 24 mg ha^−1^ bentonite had the highest grain protein, fat and fiber content while in last the two years, 24 and 30 mg ha^−1^ bentonite had the highest grain protein, fat and fiber content. The grain protein, fat and fiber content increased by up to 28%, 10% and 20% respectively over the control without bentonite from 2011 to 2015.

### Correlations among soil and crop parameters

There were highly significantly positive correlations among emergence rate, soil available water, field water capacity, photosynthesis parameters and grain quality parameters. Correlation coefficients ranged from 0.91 to 0.99 (Table 5).

**Table 5:** Correlation among all the parameters.

Index	Emergence rate	Above-ground dry matter	SPAD	Pn	Tr	WUE	Soil available water	Field water capacity	Fat	Protein	Fiber
Emergence rate	1										
Above-ground dry matter	0.956**	1									
SPAD	0.993**	0.977**	1								
Pn	0.993**	0.948**	0.991**	1							
Tr	0.987**	0.947**	0.981**	0.993**	1						
WUE	0.990**	0.948**	0.992**	0.995**	0.976**	1					
Soil available water	0.986**	0.967**	0.985**	0.978**	0.966**	0.982**	1				
Field water capacity	0.984**	0.958**	0.985**	0.993**	0.989**	0.986**	0.986**	1			
Fat	0.956**	0.993**	0.971**	0.935**	0.933**	0.938**	0.965**	0.940**	1		
Protein	0.934**	0.995**	0.958**	0.920**	0.914**	0.926**	0.960**	0.937**	0.991**	1	
Fiber	0.969**	0.992**	0.990**	0.967**	0.959**	0.969**	0.964**	0.963**	0.982**	0.977**	1

**Significant at 0.01 level. Pn: net photosynthetic rate; Tr: transpiration rate; SPAD: leaf soil plant analysis development; WUE: leaf water use efficiency.

## Discussion

### Field water holding capacity (FC) and plant available water (PAW)

Millet heading stage appeared around 90 d after sowing, and large quantities of soil water and nutrients were required to meet vigorous growth demands of millet at this stage. When mixed into soil by tillage, polymer superabsorbent soil amendments can quickly absorb a large amount of water under sufficient water conditions, and then slowly release water for plant uptake under drought conditions^43,44^. Our study showed that the effect of bentonite extended deeper into the 0–40 cm layer with increasing time since initial application, likely due to more uniform mixing with annual tillage. Our results indicated that addition of bentonite increased retention of soil water and soil available water up to 12% and 10% respectively in the surface soil layers (Figs.1 and 2), which would be available for crop use. This can improve crop production in semi-arid regions with limited rainfall, and alleviate the need for irrigation. The bentonite can absorb a large amount of water and hold it within the bentonite crystal structure^45^. In addition, the bentonite (75 μm particle size) was mixed intimately with sandy soil and created smaller pores which can retain more water. Suzuki and Noble also reported that the application of bentonite amendment to a sandy soil over two years increased the available water for crop growth which was attributed to an increase in porosity and altered pore size distribution^46^. Our result agreed with a report that when clay with different aggregate sizes was added to a sandy soil simulating clay delving from a clay rich subsoil, plant available water and water retention capacity increased in proportion to the amount of clay added, and in particular, application of smaller clay aggregates (≤ 6 mm) had a greater effect^47^. Another study also reported that available water content increased by 6.7–13.3% in calcareous sandy soils after bentonite application^48^. In contrast, another study observed that water absorbing soil amendments had a negative effect on crop performance for maize, possibly because they held the water too tightly, limiting water uptake by crops under limited water conditions^49^. The disagreement among studies may due to differences in amendment type and application rate. The mechanism by which water is held by bentonite, a natural clay material, and synthetic superabsorbent polymers is very different which results in different optimum application rates, and different water release characteristics. A previous seven-year study reported that a single application of bentonite-humic acid (20 mg ha^−1^) to sandy soil increased soil water storage and maize yield in a semi-arid region, and it had the greatest effect in the first five years following application^50^. Another study reported that large amount of superabsorbent resin quickly absorbed water, but the water was tightly held by the molecular structure and could not be released fast enough for plant use^51^. Other reasons may be due to differences in soil and external environmental factors such as potential evapo-transpiration.

### Millet growth and photosynthesis

The water stored in the soil before sowing and during the growing season plays an important role in crop production under limited water supply^52^. Insufficient stand establishment of crop is a major problem in the region along the Great Wall in Inner Mongolia in northern China which is a semi-arid area, and is a critical factor affecting crop yield^53^. Our data showed that bentonite amendment improved millet emergence rate (ER), AGDM at different days after sowing from 2011 to 2015 and photosynthesis parameters at 90 d after sowing (Pn, Tr, SPAD and WUE) (Figs. 3–5) from 2013 to 2015. This is comparable to other research that showed application of superabsorbent polymer significantly improved photosynthesis characteristics^54^, maize yield and total dry matter under drought stress conditions^55^. We attributed this to improved soil water storage and availability of nutrients for crop growth by retaining limited rainfall and reducing loss of water from the soil^56^, thereby alleviating the drought stress in the crop. In addition, the application of bentonite with calcium, magnesium and potassium (5, 10, 20, 40 mg ha^−1^) could increase plant biomass by increasing cation exchange capacity (CEC) and making more exchange sites available to hold plant nutrients for plant growth^57^. Nutrient retention by bentonite might also contribute to improvements in crop performance, but our experiment did not address this aspect. In our research, K, mg and Fe contained in the bentonite could contribute to increased crop growth, and improve photosynthesis, and thus enhance AGDM accumulation. Magnesium is major element in chlorophyll and some of the observed photosynthesis response to bentonite might be a result of additional magnesium in the bentonite. The effects of bentonite water absorbing properties, and potassium and micronutrients on crop growth parameters are confounded in this experiment; further research is needed to separate the soil water holding aspects of bentonite from the chemical effects of potassium and other micronutrients contained within the bentonite. Previous studies showed that drought conditions have a negative effect on photosynthesis processes which affected carbon assimilation and growth by closure of plant leaf stomates and reduced permeability of mesophyll cells^15,58^.

Combining our published results on ER and AGDM from the first three years^34,35^ and the present results from the latter two years for the same experiment, it was evident that the amendment effect on millet emergence rate increased with time, and the photosynthesis characteristics and emergence rate had similar trends. These may be due to improvement in soil structure by bentonite with time as bentonite is stable in soil whereas some other synthetic superabsorbent polymer soil amendments break down over time. Also this may be due to the capacity of bentonite to intercalate fixed soil carbon and volatile groups over time by metal oxides, establishing a microbial and nutrient reservoir^59^. The improvement in AGDM by the amendments was greatest during the early growing season when millet was growing rapidly, the demand for water was high and rainfall was low.

### Grain quality

Millet is a tropical cereal grain used for forage and food, and is very diverse in terms of grain structure and chemical composition. It has been cultivated to produce traditional products such as porridges, flatbreads and beers^60^**.** In this study, the bentonite increased grain protein, fat and fiber content in all five years (Fig. 6) which would improve the grain quality for the above food products. This is similar to the results of another study that bentonite amendments can improve sugar beet quality and increase sugar beet yield^61^. The amended soil retained larger amounts of water and nutrients than soil without amendment. This allows the absorbed water and nutrients to be held for a longer time and used by the crop when drought occurs thereby improving crop performance^62,63^. Grain protein for all rates of bentonite was significantly (*P* < 0.05) higher than the control without bentonite in all five years except for 2014. This might be explained by more even rainfall throughout the 2014 growing season: the positive effect of the amendments is derived from their ability to hold water during periods of intermittent rainfall, but under even and sufficient rainfall, this benefit is not realized.

The combined effect of improved yield and bentonite cost was reported in our previous publication^31^. Compared with control without bentonite, 6, 12, 18, 24 and 30 mg ha^−1^ bentonite increased total grain yield over five years by 614, 1795, 3528, 3613 and 3041 kg ha^−1^ respectively, and based on a bentonite cost in China of 63 US $ Mg^−1^, improved respective total net return over five years by 64, 536, 1406, 1089 and 300 US $ ha^−1^.

In semi-arid and arid areas, soil is characterized by low water-holding capacity, low fertility and low production. Some studies found that water absorbing soil amendments reduced soil erosion, improved soil nutrients and water-holding capacity and available water and nutrients for crop uptake, and thereby reduced the environmental pollution and enhanced soil productivity^64,65^. Our results suggest that bentonite soil amendments can also improve crop performance attributes and contribute to sustainable agricultural development in arid and semi-arid regions.

## Conclusions

Bentonite amendments increased field water holding capacity and plant available water at 90 d after sowing in all three years (2013–2015). Bentonite significantly (*P* < 0.05) increased millet emergence rate, aboveground dry matter accumulation, photosynthesis parameters (Pn, Tr, SPAD and WUE) and grain quality parameters (protein, fat and fiber). The 24 mg ha^−1^ bentonite amendment had the greatest effect on crop performance parameters averaged over five years, and on photosynthesis parameters averaged over the three years (2013–2015) that they were measured; the 18 mg ha^−1^ bentonite amendment rate had the greatest effect on grain quality. Bentonite, which is plentiful in China, is a stable mineral requiring only one application and therefore has a distinct advantage over synthetic water absorbing polymers which break down over several years and must be periodically reapplied to maintain their effect. Thus, application of bentonite may be a practical and effective strategy for improving millet production in semi-arid regions in northern China or the regions with a similar environment.
